# Deep learning model using contrast-enhanced CT imaging features and clinical variables to predict surgical risk after hepatic resection for hepatocellular carcinoma

**DOI:** 10.3389/fonc.2026.1910921

**Published:** 2026-09-03

**Authors:** Kimika Kato, Yasuhito Mitsuyama, Hiroji Shinkawa, Takuma Okada, Atsushi Sugimoto, Ryota Tanaka, Sadaaki Nishimura, Jun Tauchi, Masahiko Kinoshita, Genki Watanabe, Kohei Nishio, Takeaki Ishizawa

**Affiliations:** 1 Department of Hepatobiliary–Pancreatic Surgery, Osaka Metropolitan University Graduate School of Medicine, Osaka, Japan; 2 Department of Artificial Intelligence, Osaka Metropolitan University Graduate School of Medicine, Osaka, Japan

**Keywords:** artificial intelligence, deep learning, hepatocellular carcinoma, intraoperative blood loss, postoperative complications

## Abstract

**Background:**

Accurate preoperative risk assessment is crucial for patients undergoing hepatic resection for hepatocellular carcinoma (HCC). Conventional prediction methods are limited by the inability to capture complex relationships among multiple factors, and existing deep learning (DL) approaches focus on individual surgical outcomes. In this study, we developed and evaluated a DL model that integrates preoperative contrast-enhanced computed tomography (CT) imaging features and clinical variables to predict intraoperative blood loss and major postoperative complications.

**Methods:**

We analyzed data of 622 patients who underwent initial hepatectomy for solitary HCC between 2006 and 2021. Patients were randomly assigned to training (n = 498), validation (n = 62), and test (n = 62) cohorts. Combining convolutional neural networks and multilayer perceptron architectures, the DL model evaluated both intraoperative blood loss and postoperative complication risks. Patients were classified into major and minor intraoperative blood loss groups. Postoperative complications were defined as events of Clavien–Dindo grade IIIa or higher. Model performance was assessed via the area under the receiver operating characteristic curve (AUC).

**Results:**

For predicting major intraoperative blood loss, the model achieved AUCs of 0.81 and 0.80 in the validation and test cohorts, respectively. Furthermore, predicted blood loss significantly correlated with actual blood loss (Spearman’s ρ = 0.500, p < 0.001). For predicting postoperative complications, the model demonstrated AUCs of 0.65 in the validation cohort and 0.63 in the test cohort. Patients stratified as high risk exhibited significantly higher complication rates than those in the low-risk group across both cohorts.

**Conclusions:**

The integrated DL model enabled the simultaneous prediction of intraoperative blood loss and postoperative complications. This framework may contribute to comprehensive preoperative surgical risk assessment, although further validation is required to confirm its clinical utility.

## 1 Introduction

Liver resection is a standard curative treatment for hepatocellular carcinoma (HCC) and other hepatic malignancies; however, perioperative risks remain a major clinical concern. In particular, intraoperative blood loss and postoperative complications are closely associated with prolonged hospitalization, posthepatectomy liver failure (PHLF), and increased mortality (1–4). Therefore, accurate preoperative assessment of surgical risk is essential for optimizing operative strategies and perioperative management.

Several clinical factors, including liver function, tumor characteristics, and patient comorbidities, have been reported as predictors of surgical risk (5, 6). However, accurate preoperative risk assessment remains challenging because of the complex and heterogeneous nature of patient conditions. Although conventional statistical models and clinical scoring systems provide useful information for surgical risk evaluation (7, 8), their predictive performance may be limited in certain patients due to the difficulty in capturing complex relationships among multiple factors.

In clinical practice, intraoperative blood loss and postoperative complications represent different but complementary aspects of surgical risk. Blood loss reflects technical and anatomical complexity during surgery, whereas postoperative complications are influenced by both surgical invasiveness and patient-related factors. In addition, excessive intraoperative blood loss is associated with a higher incidence of postoperative complications and poorer postoperative recovery (9). Therefore, a framework that can simultaneously evaluate both these factors using preoperative information may provide a more comprehensive assessment of patient risks.

Recently, artificial intelligence-based approaches, particularly deep learning (DL) models, have shown promise in medical image analysis. Convolutional neural networks (CNNs) enable automatic extraction of complex features from computed tomography (CT) images and have demonstrated potential in predicting surgical outcomes (10–12). Previous studies have reported models for predicting intraoperative blood loss using machine learning approaches (13, 14), as well as models for predicting postoperative complications, including PHLF, using imaging and clinical data (15–17). In particular, Famularo et al. developed machine-learning models integrating multiphasic CT-based radiomics features and clinical variables for the preoperative prediction of PHLF after hepatectomy for HCC (17). However, these outcomes have generally been evaluated separately, and their combined assessment remains insufficient for comprehensive surgical risk evaluation.

In this study, we aimed to develop a DL model integrating preoperative CT imaging features and clinical variables to predict intraoperative blood loss and postoperative complications in patients undergoing hepatectomy, with the goal of developing a clinically useful preoperative predictive model for surgical risk.

## 2 Materials and methods

### 2.1 Data source

We used data of patients who underwent initial hepatectomy for HCC at Osaka Metropolitan University between January 2006 and December 2021. Patients were randomly divided into training, validation, and test cohorts. There was no overlap of patients among the three datasets.

This study was conducted in accordance with the Declaration of Helsinki and approved by the institutional review board (approval No. 3166). The requirement for informed consent was waived due to the retrospective study design and available opt-out option. The study was conducted and reported in accordance with the Transparent Reporting of a multivariable prediction model for Individual Prognosis Or Diagnosis (TRIPOD) statement.

### 2.2 Clinical variables

Clinical variables were collected from medical records and included age, sex, body mass index, history of diabetes mellitus, alanine aminotransferase level, platelet count, Child–Pugh class, American Society of Anesthesiologists physical status class, surgical approach (laparoscopic or open), and type of hepatectomy (major hepatectomy [≥ 3 segments] or minor hepatectomy [< 3 segments]).

### 2.3 CT image processing

Preoperative contrast-enhanced CT images were acquired in the axial plane with a 1.0-mm slice thickness. Arterial-phase CT images were used for model development, and a single axial image depicting the largest tumor diameter was extracted for DL analysis.

Images were used as one-channel input images. Candidate image sizes of 256, 320, and 512 pixels were evaluated during preprocessing and hyperparameter search. For each candidate image size, the longer side of the image was resized to the target size while preserving the aspect ratio, and the shorter side was padded with black pixels. RandAugment was applied for data augmentation during model training (18).

### 2.4 Outcomes

The primary outcomes of this study were intraoperative blood loss and postoperative complications. Intraoperative blood loss was evaluated both as a continuous and a categorical variable. For model development, intraoperative blood loss was dichotomized based on the median value in the training cohort, and patients were classified into major and minor blood loss groups.

The Clavien–Dindo classification was used to grade the severity of all postoperative complications occurring within 30 days after surgery (19). In this study, postoperative complications were defined as events of Clavien–Dindo classification grade IIIa or higher. Bile leakage (20) and PHLF (21) were defined according to the International Study Group of Liver Surgery. Surgical site infection was defined as an infection at any surgical incision or organ/space accessed during surgery according to the guidelines provided by the Centers for Disease Control and Prevention (22).

### 2.5 Model development

**Figure 1:**
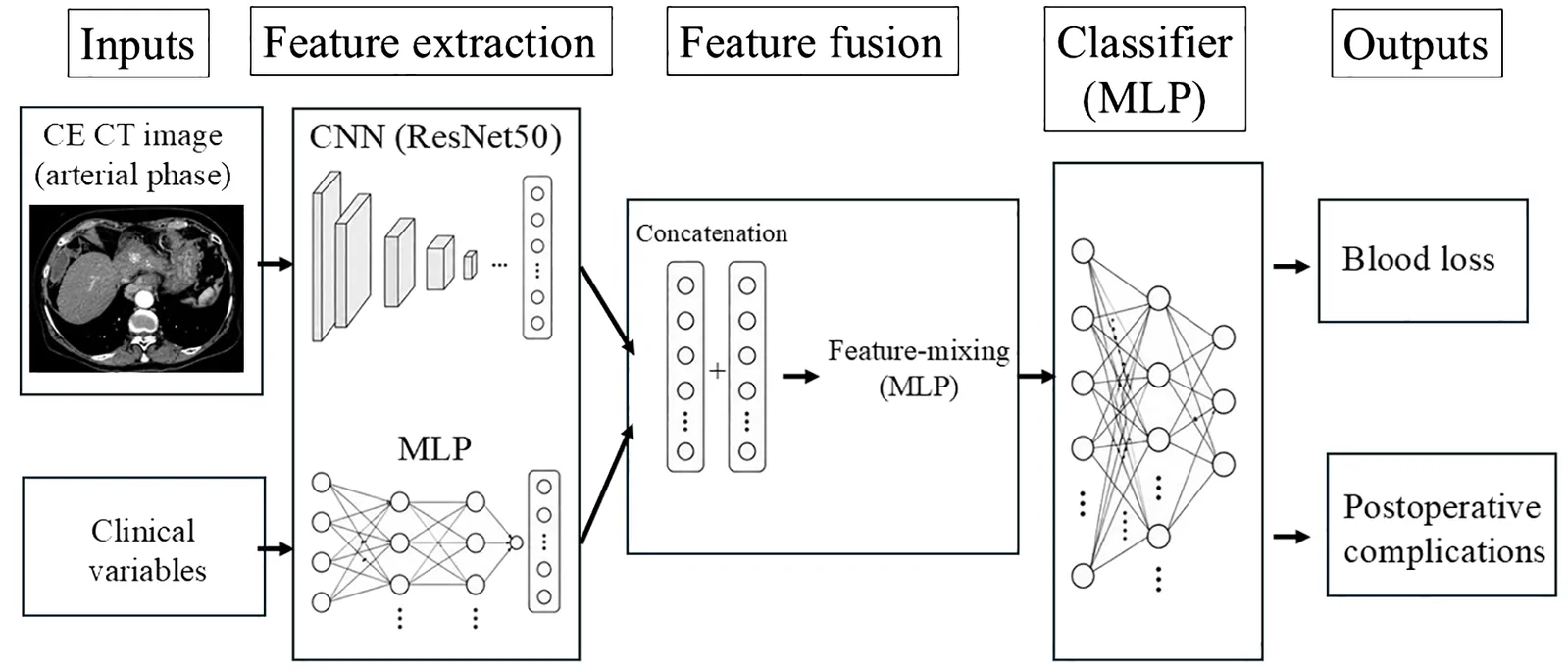
Schematic overview of the proposed multimodal classification framework. Contrast-enhanced (CE) computed tomography (CT) images were processed using a convolutional neural network (CNN) feature extractor (ResNet50) to generate imaging features. In parallel, preoperative clinical variables, including age, sex, body mass index, diabetes mellitus, alanine aminotransferase, platelet count, American Society of Anesthesiologists Physical Status classification, Child–Pugh classification, type of hepatectomy, and surgical approach, were processed using a multilayer perceptron (MLP) to generate clinical features. The extracted imaging and clinical features were concatenated, processed by a feature-mixing MLP, and subsequently passed to an MLP classifier to simultaneously predict intraoperative blood loss (≥ median) and postoperative complications (Clavien–Dindo grade ≥ IIIa).

Preprocessed preoperative contrast-enhanced CT images and the above-listed clinical variables were used to develop the multimodal DL model for predicting higher intraoperative blood loss and postoperative complications (10, 23). The model was implemented as a two-label classification model. CT images were processed by a CNN branch and clinical variables by a multilayer perceptron (MLP) branch. Candidate CNN architectures included ResNet50 (24), InceptionV3 (25), and DenseNet161 (26). The image-derived features and MLP-derived clinical features were concatenated and passed through a feature-mixing MLP to the final classifier (**Figure 1**). The final adopted architecture was MLP+ResNet.

The model was implemented using PyTorch (27) and the Nervus source code (https://github.com/Medical-AI-Lab/Nervus) (28). A pretrained model was used for the CNN branch, consistent with the final saved setting. Stochastic gradient descent (learning rate, 0.05–0.001 for learning rate), Adam (default parameters), and Adagrad (default parameters) were evaluated as candidate optimizers. The final model was trained using cross-entropy loss and the Adam optimizer (29) with a learning rate of 0.001. Batch size was evaluated from 16 during hyperparameter search, and the final saved setting used a batch size of 128. Training was performed for 50 epochs. The best checkpoint was selected according to the lowest total validation loss.

### 2.6 Model evaluation

Model performance was evaluated using the independent test dataset. Receiver operating characteristic (ROC) curves were constructed for predicting intraoperative blood loss and postoperative complications using the predictive values generated by the DL model, and the corresponding area under the curve (AUC) values were calculated. For the independent test dataset, 95% confidence intervals (CIs) for the AUCs were also calculated. Optimal cutoff values were determined using the Youden index.

For comparison, clinical-only logistic regression models were constructed using the same preoperative clinical variables included in the multimodal DL model. Their predictive performance was evaluated in the independent test dataset using ROC curve analysis, and the corresponding AUCs with 95% CIs were calculated.

In addition to binary classification, the predictive values for intraoperative blood loss were treated as continuous variables to assess their relationship with actual intraoperative blood loss. We analyzed the correlation between the predictive values and actual blood loss using Spearman’s rank correlation coefficient.

Additionally, representative Grad-CAM++ saliency maps were generated from the arterial-phase CT images to visualize the image regions contributing to the model predictions for intraoperative blood loss and postoperative complications (30).

### 2.7 Feature importance

Importance values for each explanatory variable included in the DL model were calculated using permutation importance (31). Permutation feature importance is a model inspection technique that is particularly useful for nonlinear or opaque estimators, rendering a decreased model score when a single feature value is randomly shuffled. This procedure breaks the relationship between the feature and target; thus, the drop in the model score is indicative of how much the model depends on the feature.

### 2.8 Statistical analysis

The Kruskal–Wallis and chi-square tests were used to compare continuous and categorical variables, respectively. We employed ROC curve analysis to evaluate the predictive performance of differential models. The Youden index, which was calculated by ROC curves, was used to discriminate between high and low risk for postoperative complications.

All statistical analyses were performed using EZR software (Saitama Medical Center, Jichi Medical University, Saitama, Japan). P-values less than 0.05 were considered to indicate statistical significance.

## 3 Results

### 3.1 Patient characteristics

A total of 622 patients with solitary HCC were included, divided into training (n = 498), validation (n = 62), and test (n = 62) cohorts. The demographics and surgical outcomes of the overall cohort are shown in **Supplementary Table 1**. The median age of the study cohort was 71 years, and 21 patients were classified into Child–Pugh class B. The median tumor diameter was 2.8 cm. Partial liver resection, segmentectomy, sectionectomy, bisectionectomy, and trisectionectomy were performed in 375, 61, 105, 77, and 4 patients, respectively. The median intraoperative blood loss was 257.5 g (interquartile range, 75–713.8 g). Postoperative complications of Clavien–Dindo grade IIIa or higher occurred in 79 patients (12.7%).

The characteristics of patients in the training, validation, and test cohorts are shown in **Table 1**. The median blood loss was 250 g in the training cohort, 367.5 g in the validation cohort, and 260 g in the test cohort. Postoperative complications were observed in 60 (12%), 8 (13%), and 11 (18%) patients in the training, validation, and test cohorts, respectively.

**Table 1:** Patient characteristics across the training, validation, and test cohorts.

Variables	Training cohort (n = 498)	Validation cohort (n = 62)	Test cohort (n = 62)	p-value
Sex, male/female	369/129	47/15	45/17	0.92
Age, years	71 (64–75)	73 (67–76)	71.5 (67–77)	0.09
BMI, kg/m²	23.4 (21–25.8)	22.4 (20.1–24.8)	24.0 (21.3–25.6)	0.31
Diabetes mellitus	169 (33.9%)	25 (40.3%)	24 (38.7%)	0.50
ALT, IU/L	27 (18–40)	31 (17–51)	29 (21–42.8)	0.46
AFP, ng/mL	8.5 (3.9–40.1)	9.4 (3.4–168.2)	6.2 (3.8–52.2)	0.80
Platelet count, ×10^4^/μL	15.0 (11.3–19.1)	16.1 (12.5–21.4)	14.8 (11.3–20.3)	0.22
Tumor diameter, cm	2.7 (1.9–4.3)	4.0 (2.5–5.4)	2.5 (1.8–4.0)	0.0035
Child–Pugh class A/B	461/37	60/2	60/2	0.99
ASA-PS class 3 or 4	77 (15.5%)	12 (19.4%)	11 (17.7%)	0.68
Laparoscopic approach	247 (49.6%)	26 (41.9%)	28 (45.2%)	0.45
≥Bisectionectomy	58 (11.6%)	11 (17.7%)	10 (16.1%)	0.28
Operative time, min	271.5 (200–354)	281 (236–359)	289 (200–377)	0.43
Intraoperative blood loss, g	250 (75–700)	367.5 (117.5–882.5)	260 (57.0–625)	0.14
Postoperative complication (Clavien–Dindo grade ≥IIIa)	60 (12%)	8 (12.9%)	11 (17.7%)	0.45
Posthepatectomy liver failure, grade B/C	33 (6.6%)	1 (1.6%)	9 (14.5%)	0.016

Data are presented as numbers (percentage) or median (interquartile range).

ALT, alanine aminotransferase; AFP, alpha-fetoprotein; BMI, body mass index; ASA-PS, American Society of Anesthesiologists physical status.

### 3.2 Model development

Each model was independently developed using the training dataset and tuned with the validation dataset for 100 training epochs. The loss value on the separate validation dataset was then used to determine the model performance. The final optimizer for all models was Adam (learning rate = 0.001) with a batch size of 64. The best-performing models were obtained with an image size of 512 pixels in both the image-based and mixed models; ResNet was the best-performing CNN architecture.

### 3.3 Model performance

**Figure 2:**
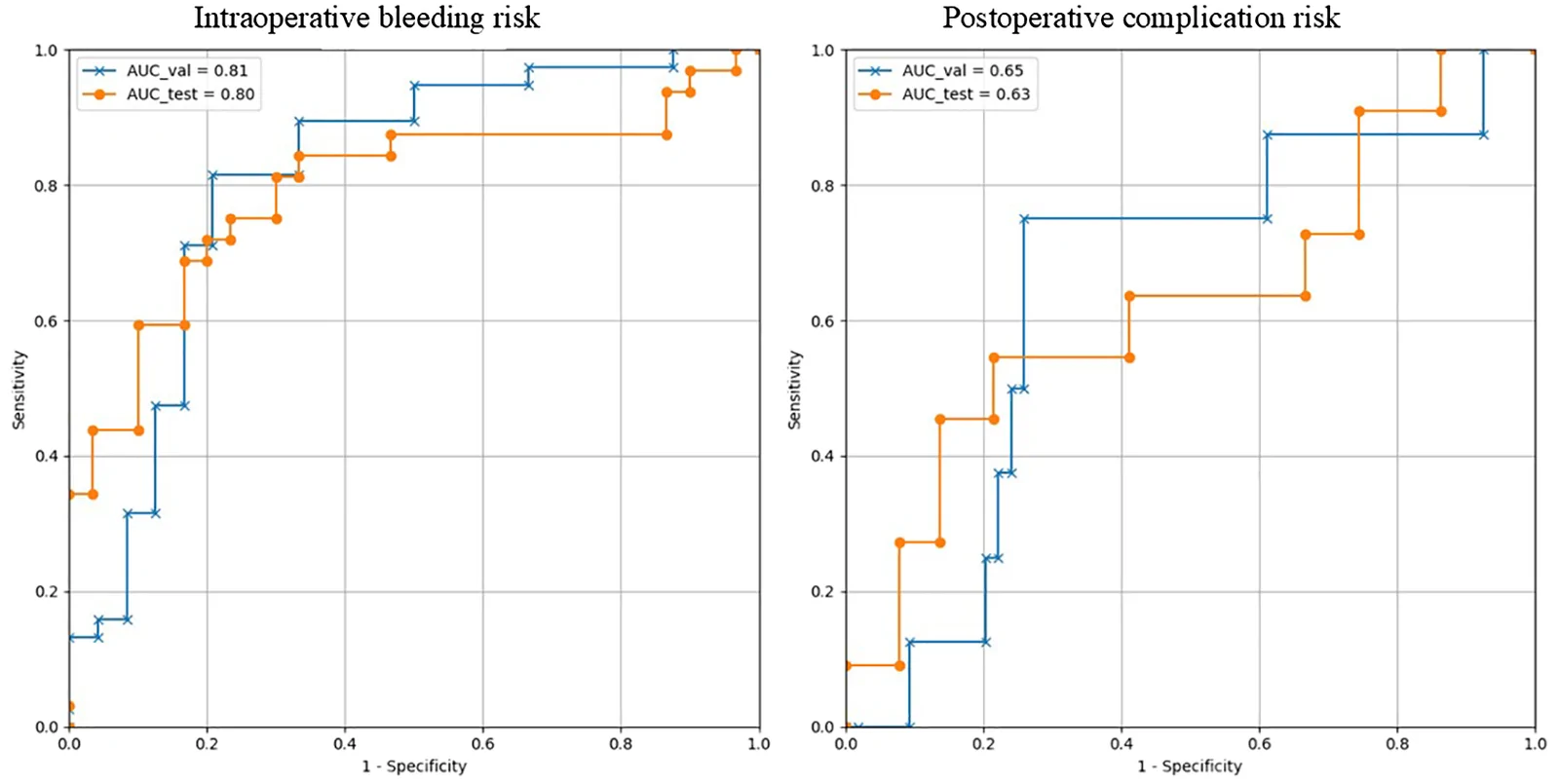
Model performance analysis. Receiver operating characteristic curves for the validation (blue) and test (orange) datasets for predicting intraoperative blood loss and postoperative complication risks. Note: AUC, area under the receiver operating characteristic curve.

For predicting intraoperative blood loss ≥250 g, the DL model achieved AUC values of 0.81 in the validation dataset and 0.80 (95% CI, 0.68–0.91) in the test dataset. The respective optimal cutoff values were 0.006 and 0.523. For predicting postoperative complications (Clavien–Dindo grade ≥IIIa), the model demonstrated moderate performance, with AUC values of 0.65 in the validation dataset and 0.63 (95% CI, 0.43–0.83) in the test dataset (**Figure 2**). The optimal cutoff values for postoperative complications were –1.545 and –1.453, respectively. Using the optimal cutoff in the test cohort, the model achieved an accuracy of 74.2%, sensitivity of 54.5%, specificity of 78.4%, precision of 35.3%, and an F1-score of 42.9% (**Supplementary Table 2**).

**Supplementary Figure 1** presents representative arterial-phase CT images together with the corresponding Grad-CAM++ saliency maps. The saliency maps highlighted the tumor and adjacent vascular structures, providing visual insight into the image regions contributing to the model predictions.

### 3.4 Feature importance

**Figure 3:**
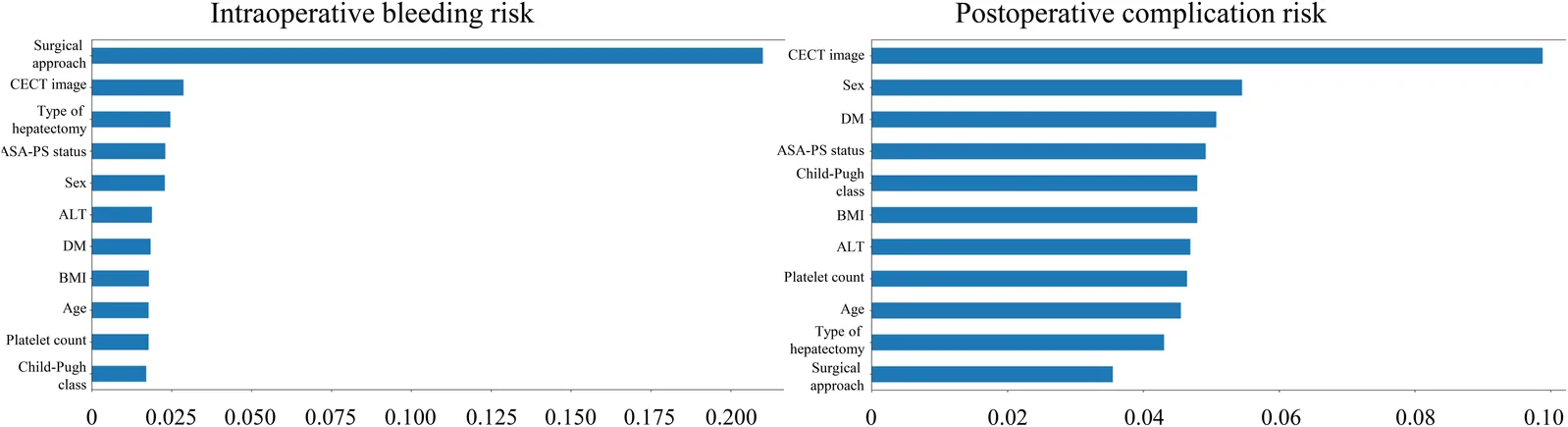
Permutation importance analysis. The graph bars show the importance of each explanatory variable for the prediction of the deep learning model. A large positive value indicates the feature is very relevant to detect positive output. CECT, contrast-enhanced computed tomography; ALT, alanine aminotransferase; BMI, body mass index; DM, diabetes mellitus; ASA-PS, American Society of Anesthesiologists physical status.

Permutation importance analysis demonstrated that both CT imaging features and clinical variables contributed to model performance. Among all variables, surgical approach and CT imaging features showed relatively high importance in predicting both intraoperative blood loss and postoperative complications (**Figure 3**).

### 3.5 Correlation between predicted and actual blood loss

**Figure 4:**
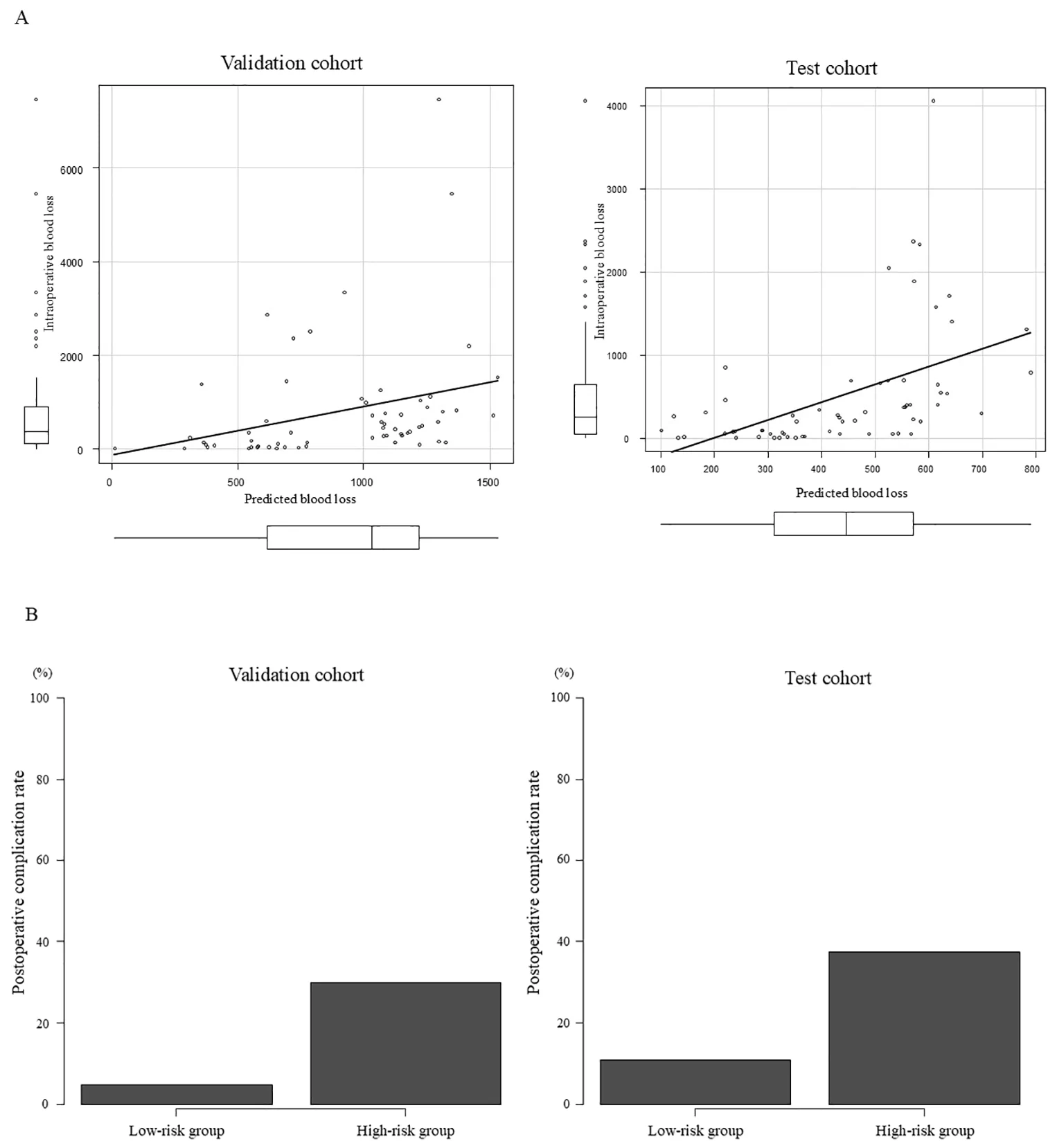
Additional analyses. **(A)** Correlation between predicted and actual intraoperative blood loss in the validation and test cohorts. **(B)** Postoperative complication rates in the high- and low-risk groups in the validation and test cohorts stratified according to the predictive model.

The predicted blood loss showed a significant correlation with the actual intraoperative blood loss (Spearman’s rank correlation coefficient: ρ = 0.500, p < 0.001 in the validation cohort; ρ = 0.623, p < 0.001 in the test cohort) (**Figure 4A**).

### 3.6 Postoperative complication rates stratified by surgical risk

Based on the cutoff values of the predictive model for postoperative complication risk, patients were classified into high- and low-risk groups. In the validation cohort, postoperative complications occurred in 6 (30%) patients in the high-risk group and 2 (4.8%) patients in the low-risk group (p = 0.011). Similarly, in the test cohort, postoperative complications occurred in 6 (37.5%) patients in the high-risk group and 5 (10.9%) patients in the low-risk group (p = 0.026) (**Figure 4B**).

In addition, based on the cutoff values of the predictive model for intraoperative bleeding risk, patients were classified into high- and low-risk groups. In the test cohort, PHLF grade B occurred in 6 (26%) patients in the high-risk group and 3 (8.3%) patients in the low-risk group (p = 0.15).

### 3.7 Comparison with a clinical-only logistic regression model

To evaluate whether CT imaging provided additional predictive value, we developed clinical-only logistic regression models using the same preoperative clinical variables. In the independent test cohort, the clinical-only model achieved an AUC of 0.80 (95% CI, 0.68–0.91) for predicting intraoperative blood loss and 0.63 (95% CI, 0.43–0.83) for predicting postoperative complications. These values were identical to the reported AUCs of the multimodal DL model.

## 4 Discussion

The proposed multimodal DL model integrating preoperative CT imaging features and clinical variables demonstrated promising performance in predicting intraoperative blood loss and postoperative complications in patients undergoing hepatic resection for HCC. The predicted blood loss showed a significant correlation with the actual intraoperative blood loss. These findings suggest that the proposed model may support the preoperative assessment of surgical risk. To the best of our knowledge, no studies to date have reported DL models using CT imaging features to predict both intraoperative blood loss and postoperative complications after resection for HCC.

Intraoperative blood loss reflects the technical and surgical complexity of liver resection, whereas postoperative complications reflect the risk of unfavorable short-term postoperative clinical outcomes (7, 8). In addition, intraoperative blood loss is closely associated with severe complications, including PHLF, as well as postoperative mortality after hepatic resection (4). Previous studies have also validated surgical difficulty scoring systems using intraoperative blood loss and postoperative complications as representative indicators of operative complexity and surgical outcomes (32, 33). In this study, although the number of patients who developed PHLF grade B/C was limited, PHLF tended to occur more frequently in patients classified as having a high predicted blood-loss risk. Taken together, simultaneous prediction of intraoperative blood loss and postoperative complications may enable a more comprehensive assessment of surgical risk than predicting either outcome alone and may therefore provide useful information for preoperative treatment planning, perioperative management, and patient counseling. Previous studies have reported predictive models for either intraoperative blood loss or postoperative complications, often focusing on a single outcome. These models were based on machine learning or DL approaches using preoperative imaging and clinical data (13–16). More recently, Famularo et al. developed machine-learning models integrating radiomics features extracted from multiphasic CT images of the non-tumoral liver with clinical variables to predict PHLF after hepatectomy for HCC (17). Their approach focused specifically on PHLF prediction using CT-based radiomics, whereas our model directly extracted imaging features from a single arterial-phase axial CT image using a CNN and integrated them with clinical variables to simultaneously predict intraoperative blood loss and postoperative complications. Simultaneous prediction of these two clinically important outcomes may provide a more comprehensive preoperative assessment of surgical risk and facilitate treatment planning and perioperative management.

CT imaging provides detailed information regarding anatomical relationships between the tumor and vascular structures, tumor location, and underlying liver characteristics, all of which are closely related to surgical complexity and intraoperative bleeding (32–34). In the present study, arterial-phase CT images were used because tumor enhancement during the arterial phase facilitates tumor identification and localization, which may provide useful information for assessing surgical complexity. Previous studies have demonstrated that anatomical factors, including proximity to major vessels and tumor location, as well as underlying liver conditions such as steatosis and cirrhosis, can influence operative difficulty and perioperative outcomes (35, 36). We have also previously reported that DL models based on preoperative CT imaging can predict postoperative recurrence and long-term prognosis after curative resection for HCC, highlighting the potential of CT-derived imaging features as clinically relevant biomarkers (11, 12). In addition, CT imaging has been reported to reflect underlying liver parenchymal conditions, including fibrosis and steatosis (37), which may affect surgical risk (38, 39). However, because the present model was developed using only a single arterial-phase axial image corresponding to the largest tumor diameter, important three-dimensional anatomical information, including vascular relationships, segmental anatomy, and liver parenchymal heterogeneity, was not fully incorporated. This technical limitation may have reduced the ability of the imaging branch to capture imaging features relevant to surgical complexity.

To further evaluate the contribution of CT imaging, we compared the multimodal DL model with clinical-only logistic regression models using the same preoperative clinical variables. In the independent test cohort, the multimodal DL model showed predictive performance identical to the reported AUCs of the clinical-only model for both intraoperative blood loss and postoperative complications. Therefore, the present study did not demonstrate a measurable incremental predictive benefit from incorporating CT imaging features into the model. Although permutation importance analysis suggested that CT-derived imaging features contributed to model prediction, this analysis does not establish an independent contribution of the imaging branch. Accordingly, the incremental value of CT imaging remains uncertain and should be evaluated in future studies using larger multicenter datasets together with more comprehensive imaging strategies, including three-dimensional and multiphasic CT data.

In the permutation importance analysis, the planned surgical approach exhibited the highest contribution to the prediction of intraoperative blood loss, which is clinically reasonable given its direct impact on bleeding risk. Because permutation importance was calculated after multimodal feature fusion, these results should be interpreted cautiously and do not necessarily indicate an independent contribution of CT imaging features.

This study has several limitations. First, the retrospective single-center design may introduce selection bias. Second, although an independent test cohort was used for model evaluation, the robustness of the proposed model should be further confirmed using larger multicenter datasets and alternative validation strategies, including cross-validation. Third, procedure-related factors, including the extent of hepatectomy, tumor location, proximity to major vascular structures, and surgical approach (laparoscopic or open), may substantially influence intraoperative blood loss and postoperative complications. However, because of the limited sample size, stratified analyses according to these procedure-related factors were not feasible. Furthermore, the model was developed based on the planned surgical approach and extent of hepatectomy determined preoperatively. Therefore, intraoperative modifications to the planned procedure were not considered and may have affected the predictive performance in some patients. Future multicenter studies including larger numbers of patients are warranted to validate the proposed model across different surgical procedures and operative complexities. Because only approximately 13% of patients underwent major hepatectomy, the present model is expected to be most applicable to surgical populations with a similar distribution of procedures. Its performance in highly complex major hepatectomy remains to be established. Fourth, the model’s predictive performance for postoperative complications was moderate, necessitating further model improvement to enhance predictive accuracy. Furthermore, the imaging branch was developed using only a single arterial-phase axial CT image rather than volumetric multiphasic CT data. Consequently, potentially important three-dimensional anatomical information relevant to surgical complexity, including vascular anatomy, liver segmental relationships, and parenchymal heterogeneity, was not incorporated into the model. Fifth, although DL models are often considered “black boxes,” we attempted to improve interpretability by performing permutation importance analysis for clinical variables and Grad-CAM visualization for CT images. Nevertheless, the biological basis underlying the learned imaging features remains difficult to fully interpret. Finally, only arterial-phase CT images were used for model development. Although arterial-phase images facilitate identification of HCC lesions, multiphasic CT imaging may provide complementary information regarding tumor characteristics and vascular anatomy. Future studies are warranted to investigate whether incorporating multiphasic CT images further improves predictive performance. In addition, the relatively small numbers of postoperative complication events in the validation and test cohorts limited the precision of the corresponding performance estimates and precluded reliable calibration analysis.

Nevertheless, because the proposed model relies solely on routinely available preoperative CT imaging and clinical variables, it may provide a practical and noninvasive framework for supporting treatment planning, patient counseling, and perioperative management in patients undergoing hepatic resection for HCC. Further multicenter validation is warranted before routine clinical implementation.

## 5 Conclusion

We developed a DL model integrating preoperative CT imaging features and clinical variables for the simultaneous prediction of intraoperative blood loss and postoperative complications. Although further validation in larger multicenter cohorts is required, this framework may contribute to comprehensive preoperative surgical risk assessment and individualized perioperative management.

## Data Availability

The raw data supporting the conclusions of this article will be made available by the authors, without undue reservation.
