# Supplementary material for: Deep learning model using contrast-enhanced CT imaging features and clinical variables to predict surgical risk after hepatic resection for hepatocellular carcinoma
Source: Front Oncol. 2026 Sep 3;16:1910921. doi: 10.3389/fonc.2026.1910921 (PMC13581739; doi:10.3389/fonc.2026.1910921)
Supplement: Supplementary file 1 [file SupplementaryFile1.docx]

Supplementary Material

# 1 Supplementary Tables

**Supplementary Table 1. Characteristics and surgical outcomes of the overall study cohort**

| Variables | |  | n = 622 |
| --- | --- | --- | --- |
| Age, years, median (IQR) | |  | 71 (65–76) |
| Sex, male/female | |  | 461 / 161 |
| BMI, kg/m², median (IQR) | |  | 23.4 (21.0–25.7) |
| Comorbidities, n (%) | |  |  |
| Diabetes mellitus | |  | 218 (35.1) |
| Hypertension | |  | 346 (55.6) |
| Dyslipidemia | |  | 124 (19.9) |
| HBsAg positive | |  | 110 (17.7) |
| HCV antibody positive | |  | 317 (51.0) |
| Laboratory data, median (IQR) | |  |  |
| Total bilirubin, mg/dL | |  | 0.6 (0.5–0.9) |
| ALT, IU/L | |  | 27 (18–42) |
| Albumin, g/dL | |  | 4.0 (3.7–4.3) |
| PT, % | |  | 95 (85–104) |
| Platelet count, ×10^4^/μL | |  | 15.1 (11.3–19.7) |
| AFP, ng/mL | |  | 8.3 (3.9–49.5) |
| Child–Pugh classification, A / B | |  | 601 / 21 |
| ASA-PS class 3 or 4, n (%) | |  | 100 (16.1) |
| Tumor diameter, cm, median (IQR) | |  | 2.8 (1.9–4.5) |
| Laparoscopic approach, n (%) | |  | 301 (48.4) |
| Bisectionectomy or greater, n (%) | |  | 79 (12.7) |
| Hepatectomy procedures, n (%) | |  |  |
| Partial resection | |  | 375 (60.3) |
| Segmentectomy | |  | 61 (9.8) |
| Sectionectomy | |  | 105 (16.9) |
| Bisectionectomy | |  | 77 (12.4) |
| Trisectionectomy | |  | 4 (0.64) |
| Operative time, min, median (IQR) | |  | 274.5 (202–356) |
| Intraoperative blood loss, g, median (IQR) | |  | 257.5 (75–713.8) |
| Postoperative complications, Clavien–Dindo grade ≥IIIa, n (%) | |  | 79 (12.7) |
| Intra-abdominal infection | |  | 37 (5.6) |
| Bile leakage | |  | 29 (4.7) |
| Pleural effusion | |  | 28 (4.5) |
| Gastrointestinal bleeding | |  | 25 (0.3) |
| Ascites | |  | 15 (2.4) |
| Wound infection | |  | 5 (0.8) |
| Intra-abdominal bleeding | |  | 2 (0.3) |
| Ileus | |  | 2 (0.32) |
| Gastrointestinal perforation | |  | 1 (0.2) |
| Arrhythmia | |  | 1 (0.2) |
| Posthepatectomy liver failure, n (%) | |  |  |
| Grade B | |  | 39 (6.3) |
| Grade C | |  | 4 (0.6) |
|  | IQR, interquartile range; ALT, alanine aminotransferase; PT, prothrombin time; AFP, alpha-fetoprotein; BMI, body mass index; HBsAg, hepatitis B surface antigen; HCV, hepatitis C virus; ASA-PS, American Society of Anesthesiologists physical status. | | |

**Supplementary Table 2. Performance metrics of the deep learning model for predicting and postoperative complications in the test cohort.**

| Outcome | AUC (95% CI) | Accuracy | Sensitivity | Specificity | Precision (PPV) | F1-score |
| --- | --- | --- | --- | --- | --- | --- |
| Higher intraoperative blood loss | 0.80 (0.678–0.913) | 0.742 | 0.656 | 0.833 | 0.808 | 0.724 |
| Postoperative complication | 0.63 (0.425–0.833) | 0.742 | 0.545 | 0.784 | 0.353 | 0.429 |

AUC, area under the receiver operating characteristic curve; CI, confidence interval; PPV, positive predictive value

# 2 Supplementary Figures

**Supplementary Figure 1.** Representative arterial-phase CT image and the corresponding Grad-CAM++ saliency heatmap. The heatmap is overlaid on the original CT image, with red indicating regions that contributed to the model prediction. The tumor and inferior vena cava are annotated for reference.
